# F19. TELOMERE SHORTENING IN YOUNG PEOPLE WITH FIRST EPISODE PSYCHOSIS: A 12-MONTH FOLLOW-UP STUDY

**DOI:** 10.1093/schbul/sby017.550

**Published:** 2018-04-01

**Authors:** David Fraguas, Sandra Recio, Covadonga M Diaz-Caneja, Maria A Blasco, Ana Carolina Moisés, Celso Arango

**Affiliations:** 1 Hospital General Universitario Gregorio Marañón; 2 Hospital General Universitario Gregorio Marañón, School of Medicine, Universidad Complutense; 3 Spanish National Cancer Research Centre (CNIO)

## Abstract

**Background:**

Short telomere length is a biomarker of cell oxidation and aging. Patients with first-episode psychosis (FEP) have been reported to have shorter telomeres than healthy controls (HC), suggesting that there is a premature and accelerated cellular aging in FEP. However, there are not data on longitudinal changes of telomere length in people with FEP relative to HC. We present preliminary results on 1-year longitudinal changes in peripheral blood mononuclear cells (PBMCs) telomere length and the proportion of PBMCs with short telomeres in young people with FEP and HC.

**Methods:**

16 young patients with FEP (43.8% female, mean age 17.9 years) and 21 young HC (61.9% female, mean age 16.6 years) were enrolled in the study. PBMCs telomere length and the proportion of PBMCs with short telomeres (i.e. <3kb) were determined using high-throughput quantitative fluorescence in situ hybridization (HT Q-FISH) at baseline (16 patients with FEP and 21 HC) and 12-month follow-up (4 patients with FEP and 4 HC).

**Results:**

At baseline, we did not find significant differences in telomere length nor in proportion of PBMCs with short telomeres between FEP patients and HC. During the one-year follow-up, we found a significantly greater loss of telomere length (p=0.019; explained variance=69.7%) and a non-significantly trend for greater increase in the proportion of PBMCs with short telomeres (p=0.097; explained variance=45.5%) in patients with FEP than in HC.

**Discussion:**

Telomere length changes during the first years of the illness can represent an early marker of accelerated cellular aging in patients with first-episode psychosis.

